# DEAE-Dextran Coated AgNPs: A Highly Blendable Nanofiller Enhances Compressive Strength of Dental Resin Composites

**DOI:** 10.3390/polym14153143

**Published:** 2022-08-02

**Authors:** Shabia Azhar, Nosheen Fatima Rana, Amer Sohail Kashif, Tahreem Tanweer, Iqra Shafique, Farid Menaa

**Affiliations:** 1 Department of Biomedical Engineering and Sciences, School of Mechanical & Manufacturing Engineering, National University of Science & Technology, Islamabad 44000, Pakistan; sazhar.bmes19smme@student.nust.edu.pk (S.A.); amer.kashif@smme.nust.edu.pk (A.S.K.); ttanweer.phd19smme@student.nust.edu.pk (T.T.); ishafique.phd21smme@student.nust.edu.pk (I.S.); 2 Integrated Nanobiotechnology Laboratory, School of Interdisciplinary Engineering & Sciences (SINES), National University of Sciences and Technology (NUST), Islamabad 44000, Pakistan; 3 Departments of Internal Medicine and Nanomedicine, California Innovations Corporation, San Diego, CA 92037, USA

**Keywords:** dental composite, mechanical strength, nanoparticles, antibacterial activity

## Abstract

Micro-crack formation and resultant bacterial infiltration are major causes of secondary caries formation in dental resin-based composite restorations. Improving dental resin composites’ mechanical and biological properties using highly bendable nanoparticles (NPs) can resolve this issue. This study aims to develop novel Diethylaminoethyl (DEAE)-Dextran silver nanoparticles (AgNPs) and subsequently modify composite resins with these NPs to enhance their mechanical and antibacterial properties. DEAE-Dextran AgNPs were successfully synthesized using a chemical reduction method that was confirmed with the help of ultraviolet-visible (UV-Vis) spectroscopy, scanning electron microscopy (SEM), Fourier-transform infrared spectroscopy (FTIR), Zeta potential, and energy-dispersive X-ray spectroscopy (EDS). Antibacterial activity of a composite disc with DEAE-Dextran AgNPs was tested against *Streptococcus mutans*, *Enterococcus faecalis,* and oral microcosm. The composite discs prepared with DEAE-Dextran AgNPs exhibited excellent antibacterial activity compared with composite resin reinforced by simple AgNPs (*p* < 0.05). Mechanical properties were significantly enhanced by adding DEAE-Dextran into composite resin (*p* < 0.05). Moreover, unlike AgNPs, DEAE-Dextran AgNPs were found to be less hemolytic. The results establish strong ground applications for DEAE-Dextran-modified dental composite resins in restorative dental applications.

## 1. Introduction

Resin-based dental composites have become popular as dental restorative materials because of their appealing clinical characteristics, such as high durability, good aesthetics, minimum toxicity, low cost, and convenience in clinical use [1]. However, resin-based composites exhibit a major limitation of polymerization shrinkage that causes residual stresses near the tooth–composite interface [2]. This leads to an increased risk of tooth– composite interfacial debonding and edge fractures, allowing bacterial entry and reinfection of dental hard tissues resulting in secondary caries [2,3]. In addition, resin-based composites lack good anti-cariogenic properties, allowing bacteria to create a biofilm on their surface [4]. For example, *S. mutans* releases acid and minimizes pH around the tooth–composite interface, leading to demineralization of tooth enamel, whereas *E. faecalis* degrades dental composite material affecting its life span [5]. Hence, there is a need to develop dental composites with enhanced mechanical strength that also possess antibacterial properties for improved longevity of resin composite restorative materials and prevent further development of secondary caries.

Nanotechnology is rapidly advancing in the realm of biomedical sciences [6,7]. In dentistry, it has played a vital role in enhancing resin materials’ physical, mechanical, and biological properties to improve service life [7,8]. Enhancement of resin composites with antibacterial inorganic filler materials has augmented a rapid progression in dental material research as they can enhance these composites’ physical, mechanical, and biological properties [9,10].

Among inorganic nanofillers, metallic nanoparticles (metallic NPs) are widely studied owing to their large surface area that increases antibacterial activity. Furthermore, metallic NPs enhance mechanical properties, such as the strength and durability of dental materials [11,12]. Among metallic NPs, silver (AgNPs) holds great importance due to its high surface area-to-volume ratio, small size, high antibacterial activity, and inexpensive synthesis [13]. Silver ions released from these NPs provide broad-spectrum antibacterial activity against Gram-positive and Gram-negative bacteria [3,14]. These properties make metallic NPs the best choice as an antibacterial nanofiller for dental composites [4,15]. Direct incorporation of AgNPs in resin composites may lead to rapid leaching of NPs and a reduction in antimicrobial properties [16]. The tendency of AgNPs to agglomerate can negatively influence performance and may restrict widespread application [16]. Polymer stabilization of AgNPs has been widely studied to minimize these limitations by enhancing the stability and blending capacity of AgNPs [17,18,19,20]. Both synthetic and natural polymers have been previously utilized to stabilize AgNPs [17,18,19,20]. They are widely studied for dental applications, such as Chitosan-AgNPs [17], lactose-modified Chitosan-AgNPs [18], Polyethylene glycol (PEG-AgNPs) [19], and Polyvinyl alcohol (PVA-AgNPs) [20]. Evidence suggests that coating NPs with a polymeric matrix enhances dispersion in resin composites and controls the quick release of antimicrobial agents [16]. Hence, resin composites modified with polymer-stabilized AgNPs can enhance dental resin composites’ mechanical and antimicrobial properties [16,21].

DEAE-Dextran is a cationic polysaccharide and a polycationic variant of carbohydrate polymeric Dextran [22]. It has been exploited in multiple biomedical applications, such as the transfer of nucleic acids into cultured human cells, RNA, or DNA transfusion, etc. It also allows long-term stable transfections [23]. DEAE-Dextran has also been used to stabilize metallic NPs such as CdS [24], gold (AuNPs) [25], and AgNPs [22]. Coating AgNPs with DEAE-Dextran is reported to decrease toxicity and improve the antibacterial activity of AgNPs [22]. Hence, they can be used as dental composite nanofiller. However, their antibacterial, mechanical, and biocompatibility in resin composites has not been studied.

This study aims to synthesize and characterize DEAE-Dextran-coated AgNPs and their incorporation into dental composite resins to enhance mechanical and biological properties. These highly bendable and stabilized NPs can improve resin-based dental restoration materials’ longevity by inhibiting micro-gaps’ development and the subsequent formation of secondary caries.

## 2. Materials and Methods

The study has been approved by the institutional review board (IRB) of the Department of Biomedical Engineering and Sciences (BMES), School of Mechanical and Manufacturing Engineering (SMME), and the National University of Sciences and Technology (NUST), Islamabad.

### 2.1. Preparation of AgNPs and DEAE-AgNPs

AgNPs were prepared by using the chemical reduction method [26]. To prepare AgNPs, 10 mL of 1 mM AgNO_3_ was added dropwise into 30 mL of NaBH_4_ solution (2 mM) with constant stirring. DEAE-AgNPs were also prepared using the chemical reduction method [22]. Subsequently, 0.51 g of AgNO_3_ and 0.17 g of DEAE-Dextran HCl powder solutions were prepared separately in 10 mL of distilled water. Both solutions were mixed with constant stirring, resulting in a milky white solution. After this, NH_3_ solution (23%) was added until the solution became clear. Later, 2.0 mM NaBH_4_ was added dropwise. All of the chemicals mentioned above were purchased from Sigma Aldrich.

### 2.2. Characterization

The DEAE-Dextran AgNPs were characterized by UV spectrophotometry (UV-2800 BMS Biotechnology Medical Services, Madrid, Spain). FTIR was performed by FTIR Spectrometer ALPHA ll (Bruker, Westborough, MA, USA). SEM and EDX were performed with SEM VEG 3 LMU (Tescan, Czech Republic), and zeta potential was analyzed by Malvern Zeta sizer (Malvern; Worcestershire, UK).

### 2.3. Modification of Composite Resin Discs

The organic polymers were used for dental composite resins Bis-GMA, UDMA, and Bis-EMA (40:40:20). AgNPs and DEAE-Dextran-coated AgNPs were incorporated into composite resins using a standardized protocol for weight percentage [27,28]. Different concentrations of DEAE-Dextran-coated AgNPs, i.e., 1%, 2.5%, and 5%, were added into composite resins and mixed for 1 min manually to uniformly distribute NPs in the composite. Discs were then prepared using a plastic mold and cured for 2 h using blue UV light of 400 mV/cm^2^ intensity with 430–480 nm wavelength.

### 2.4. Isolation of Bacterial Strains

The saliva samples were collected from 10 volunteers for bacterial isolation. Only those volunteers who had not received any dental procedures in the past were asked not to consume anything nor to brush their teeth at least two hours before the saliva collection [29]. The collected saliva samples were mixed thoroughly, serially diluted with autoclaved distilled water (1:9), and spread on Tryptic soy agar (TSA) plates with 1% sucrose. The plates were incubated for 24 h [30]. Light and dark yellow-colored colonies were picked and streaked on separate TSA plates. These colonies were streaked on blood agar plates to isolate *S. mutans* and *E. faecalis*. For the microcosm model, saliva samples were diluted using sterile glycerol up to 30% to form inoculum [30]. Serial dilutions were carried out using autoclaved distilled water. The dilutions were then utilized for antibacterial testing.

### 2.5. Antibacterial in Vitro Assay

Tryptic Soy Broth with 1% sucrose (TSBS) was used as a bacterial growth medium. From an overnight preculture of *S. mutans* and *E. faecalis*, the culture was inoculated and incubated at 37 °C. The culture was serially diluted until optical density (OD) reached 1 at 600 nm. Subsequently, 500 µL of dilution was added to each Eppendorf tube, and composite disks were immersed. The Eppendorf tubes were placed in the stand and incubated for 6 h at 37 °C. Following this, 50 µL of culture from each tube was plated on TSA plates and incubated for another 48 h. Colonies were counted manually, and log10 CFU/mL was determined. The experiments were performed in triplicates.

### 2.6. Hemolytic Activity for AgNPs and DEAE-Dextran AgNPs

A hemolytic test was performed to evaluate the cytotoxicity of the modified composites at different concentrations of DEAE-Dextran AgNPs (1%, 2.5%, and 5%). The blood samples were collected from healthy volunteers after obtaining their informed consent. The blood was centrifuged at 18,000 rpm for 10 min. The supernatant was removed and blood cells were diluted with phosphate-buffered saline (PBS) in a 1:3 ratio. The composite discs were immersed in 10 mL PBS solution and incubated for 15 min at 37 °C in a water bath. Later, 0.2 mL of the diluted blood was added into these tubes, gently inverted, and incubated for 2 h. Triton X-100 (1%) was used as a positive control and phosphate-buffered saline (PBS) was used as a negative control. After incubation, all tubes were centrifuged for 10 min at 18,000 rpm and the supernatant’s optical density (*OD*) was measured at 350 nm. The % hemolysis was calculated using the formula below [31]:

$$\%Hemolysis=\frac{Sample\;OD-Negative\;Control\;OD}{Positive\;Control\;OD-Negative\;Control\;OD}\times 100$$

### 2.7. Mechanical Testing

The compressive strength of the samples was tested after 24 h of immersion in composite discs of saliva. The compressive strength was determined using a static universal testing machine (Instron, Norwood, MA, USA) with a crosshead speed of 0.5 cm/min and a load cell of 5 KN. Samples were placed on the testing machine’s base and were subjected to compressive stress until they fractured. The force was measured in MPa [32].

$$Compressive\;strength\;\left(MPa\right)=\frac{Failure\;Load\;\left(N\right)}{Area\;\left({\mathrm{mm}}^{2}\right)}$$

### 2.8. Statistical Analysis 

Graph Pad Prism 9.2 was used for statistical analysis. Average and variance were measured and multiple group comparisons were carried out using one-way ANOVA at a significance level of 0.05.

## 3. Results

### 3.1. The Successful Synthesis of AgNPs and DEAE-Dextran AgNPs

**Figure 1:**
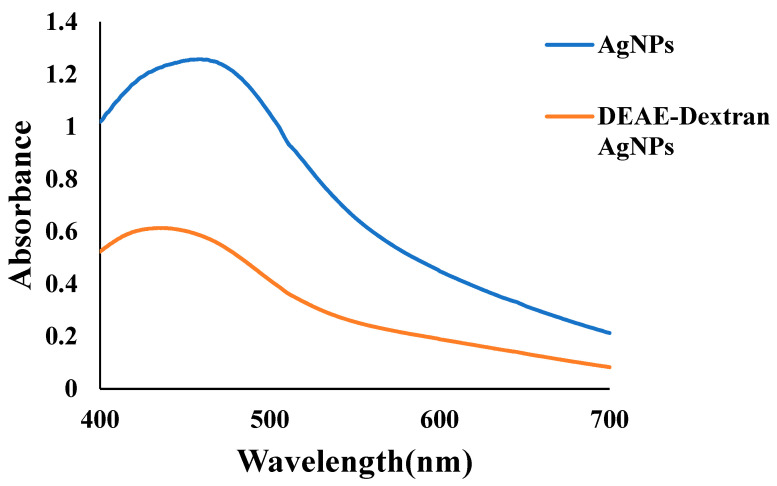
UV analysis of AgNPs and DEAE-Dextran AgNPs.

AgNPs synthesis was confirmed with the appearance of a bright yellow color. The UV peak at 412 nm confirmed the successful synthesis of AgNPs. The brown-colored solution was obtained upon coating AgNPs with DEAE-Dextran, and the peak was shifted from 412 to 420 nm. This visible peak shift showed the successful formation of DEAE-AgNPs (Figure 1).

### 3.2. Zeta Charge

The zeta potential value of AgNPs was −15.1, which changed to +16.2 with the addition of DEAE-Dextran.

### 3.3. Particle Morphology

**Figure 2:**
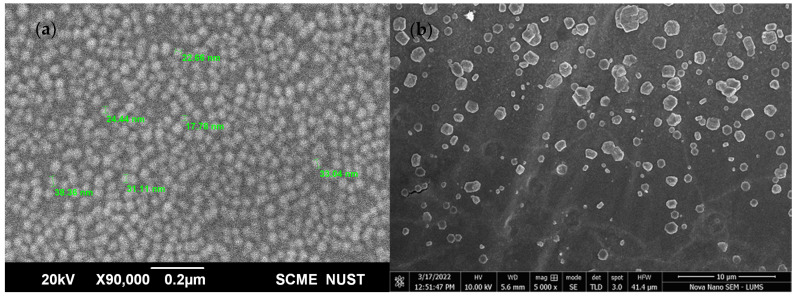
SEM images: (**a**) AgNPs and (**b**) DEAE-Dextran AgNPs.

The morphology of AgNPs and DEAE-Dextran AgNPs was analyzed with SEM at 20 kV. The SEM results revealed spherical AgNPs, whereas DEAE-Dextran AgNPs were mostly hexagonal with some exhibiting irregular shapes (Figure 2).

### 3.4. EDS Analysis

**Figure 3:**
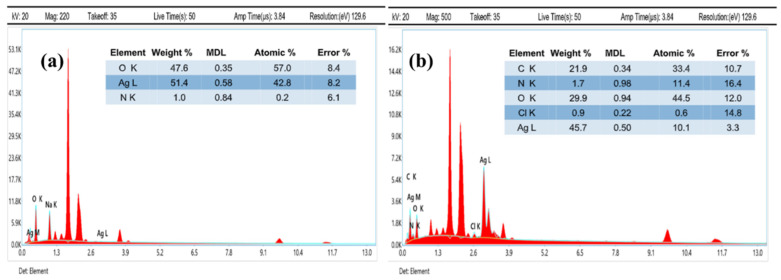
EDS analysis of: (**a**) AgNPs and (**b**) DEAE-Dextran AgNPs.

Figure 3a shows the EDS analysis of AgNPs; it showed strong silver peaks, confirming the synthesis of AgNPs. Whereas Figure 3b indicates EDS of DEAE-Dextran AgNPs, a strong peak in the carbon region and reduction in silver is observed, which is indicative of successful coating of DEAE-Dextran on AgNPs.

### 3.5. FTIR Analysis

**Figure 4:**
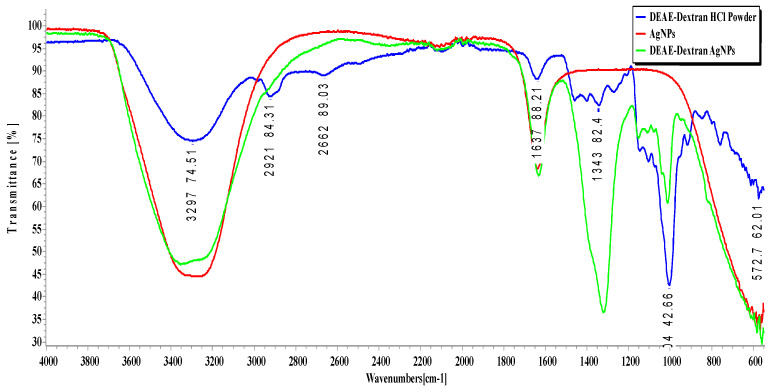
FTIR spectrum of AgNPs, DEAE-Dextran HCl, and DEAE-Dextran AgNPs.

The FTIR of AgNPs, DEAE-Dextran AgNPs, and DEAE-Dextran HCl powder is shown in Figure 4 The FTIR analysis confirmed the successful coating of DEAE-Dextran on AgNPs. A broad band surrounding 3600 cm^−1^ is assigned to OH stretching. This is due to the adsorption of water in samples that weakens the signals on a band of 2860 cm^−1^. The band at 1517 cm^−1^ represents C-O-H deformation contributing to symmetric stretching of O-C-O in the carboxylate group. The absorption of stronger bands ranges from 1188 cm^−1^ to 826 cm^−1^, indicating C-O-C vibrations. The peak of 966 cm^−1^ in the spectrum is characteristic adsorption of dextran, which shows α1, 3 glycosidic bonds. After coating, specific shifts were observed in the region above 1000 cm^−1^. The adsorption of dextran on AgNPs can be seen on 1091 cm^−1^, 1972 cm^−1^, 2008 cm^−1^, and 2161 cm^−1^ peaks that represent the stretching of DEAE-Dextran [33].

### 3.6. Antibacterial Activity of AgNPs and DEAE-Dextran AgNPs

**Figure 5:**
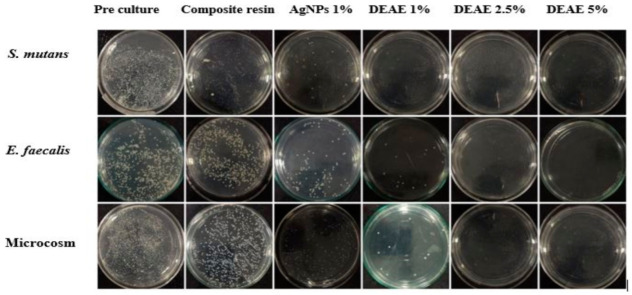
Bacterial colonies on TSA plates.

**Figure 6:**
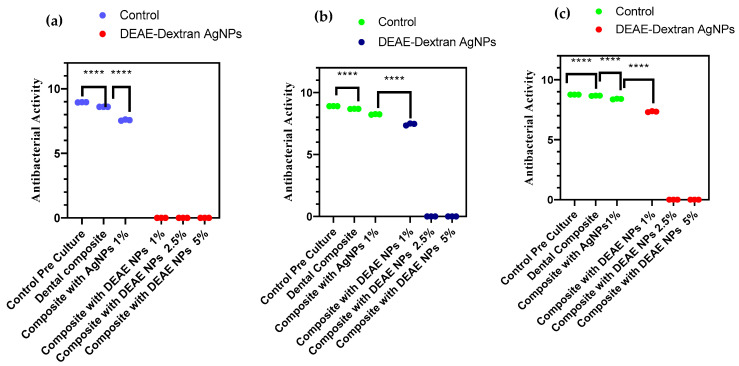
CFU/mL of: (**a**) *S. mutans*, (**b**) *E. faecalis*, and (**c**) microcosm models: antibacterial effect of modified composite resin disks (**** = *p* < 0.0001).

The results of the antibacterial activity of composites modified with AgNPs and DEAE-Dextran AgNPs are presented in Figure 5 and Figure 6, showing a comparison at different concentrations. A remarkable reduction in bacterial counts of modified composite resin discs in DEAE-Dextran AgNPs was observed compared with AgNPs modified and unmodified composite discs. The composite resins containing 1% DEAE-Dextran AgNPs inhibited *S. mutans* growth (*p* < 0.05). The *E. faecalis* and microcosm model revealed that 2.5% DEAE-Dextran AgNPs concentration was more effective than 1% Dextran (*p* < 0.05). The addition of DEAE-Dextran into composite resins significantly inhibited the growth of *S. mutans* and *E. faecalis* (Figure 6).

### 3.7. Hemolytic Assay of DEAE-Dextran AgNPs

**Figure 7:**
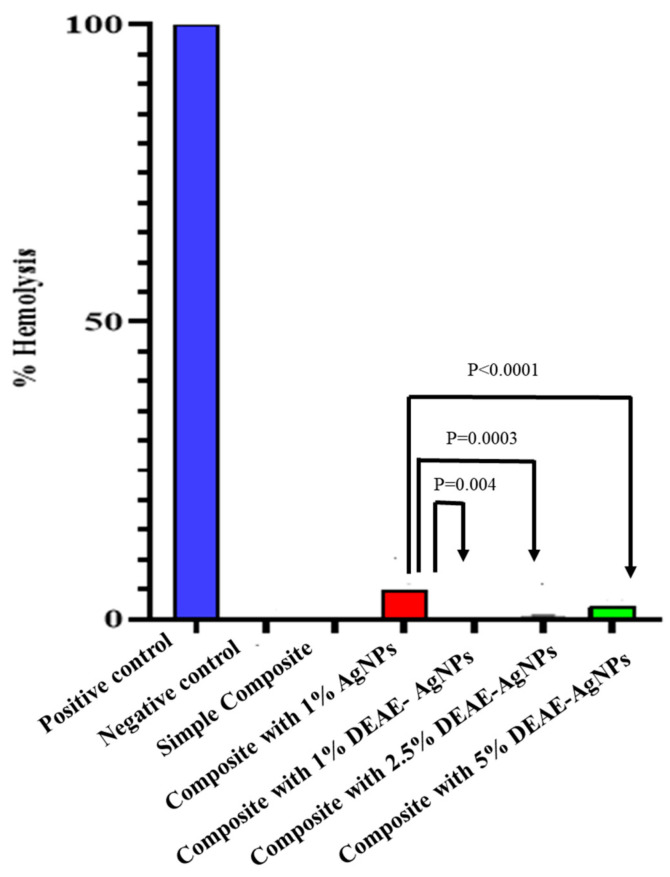
Hemolytic activity of composite resin, AgNPs, and DEAE-Dextran AgNPs. Statistically significant changes from untreated composite resin (*p* < 0.0001).

A hemolysis test was performed to determine the hemolytic effect of various dosages of DEAE-Dextran AgNPs and AgNPs in modified composite discs. The hemolytic activity of DEAE-Dextran AgNPs, AgNPs modified, and non-modified composite resin is shown in Figure 7. According to ISO/TR 7406, a 5% percentage of hemolysis is deemed safe. According to these findings, 1% AgNPs exhibited strong hemolytic activity (4.8%) compared with untreated composite resin (0%) and DEAE-Dextran AgNPs (0.2%, 0.64% and 2.08%).

### 3.8. Compressive Strength Measurement Analysis

**Figure 8:**
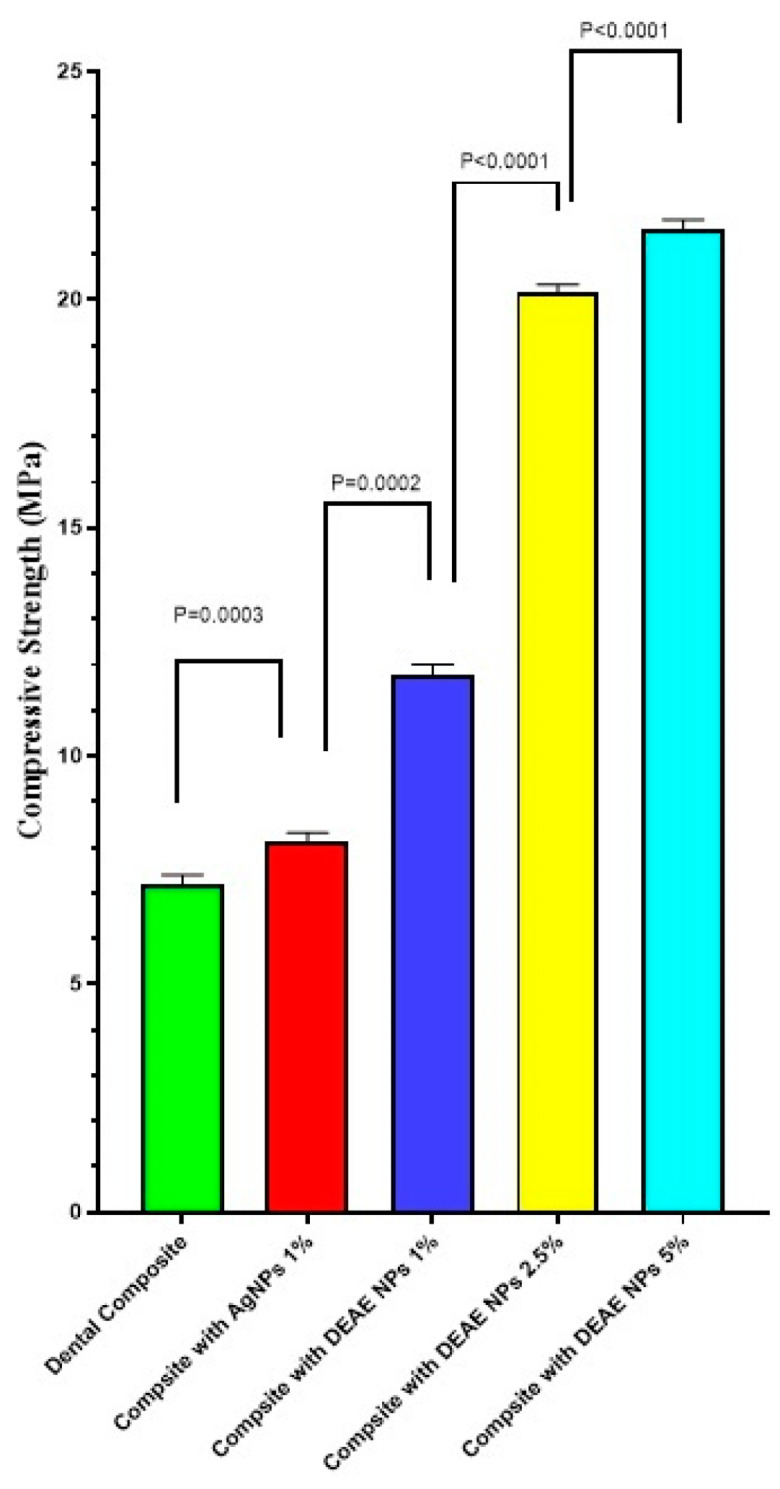
Compressive strength of modified and non-modified composite resins.

Figure 8 shows the mean compressive strength (MPa) for unmodified and modified resin composites by AgNPs and DEAE-Dextran AgNPs (wt.%). As DEAE-Dextran AgNPs were added to resin composites at 1%, 2.5%, and 5%, the compressive strength of composites resin was significantly enhanced (*p* < 0.05) compared with the unmodified group (*p* < 0.05). Composite with 5% DEAE-Dextran exhibited the highest compressive strength.

## 4. Discussion

This study presents a successful synthesis of DEAE-Dextran AgNPs and modification of resin composites with these NPs to enhance their antibacterial and mechanical properties. An enhanced blending capacity of AgNPs successfully improved the antibacterial and mechanical properties of resin composites due to the polymer coating DEAE-Dextran [21].

DEAE-Dextran, a cationic derivative of Dextran, was utilized, owing to its good biocompatibility and stabilizing capacity [22,30]. It was successfully coated on AgNPs, which UV-spectroscopy and FTIR analysis confirmed. A strong, broad UV peak of AgNPs within 400–420 nm confirmed the loading [34]. This single distinct band confirms the reduction of Ag^+^ to Ag^0^ by adding DEAE-Dextran; absorption maxima were shifted towards 450 nm, which is close to the initial peak and indicates the stability of DEAE-Dextran AgNPs [35], as seen in Figure 1 and Figure 4. During synthesis, a 3:1 molar ratio of Ag: Dex was utilized, resulting in the synthesis of irregularly shaped DEAE-Dextran AgNPs. The final composition of DEAE-Dextran NPs depended on the concentration of the reducing agent, i.e., NaBH4 [21].

Irregular hexagonal-shaped DEAE-Dextran AgNPs, almost 5 times bigger due to the coating of DEAE-Dextran, were confirmed by SEM [36]. FTIR analysis indicated peaks for amide bond stretching at 1517 cm^−1^ for DEAE-Dextran AgNPs. Some peaks in spectra were almost similar, whereas few peak shifts in the region above 1000 cm^−1^ were observed due to the coating of DEAE-Dextran on AgNPs [33]. Zeta charges upon DEAE-Dextran coating changed to +16.2, indicated stable formulation [22]. Furthermore, a positive charge on the DEAE-Dextran AgNPs enhanced antibacterial activity, as positively charged AgNPs normally cause a greater antibacterial effect than negatively charged NPs against both Gram-negative and Gram-positive bacteria [37].

For recognition as an effective nanofiller, a nanofiller should enhance antibacterial and mechanical properties, or at least should not cause any substantial changes in mechanical properties [38]. AgNPs have been previously reported as an effective antibacterial nanofiller. However, it is unstable and aggregates within the composite. Increasing the concentration of these NPs would increase toxicity and decrease the mechanical properties of composite resin [27]. It may also reduce aesthetics by imparting color to the composites. This study demonstrates that composites modified with DEAE-Dextran AgNPs show an enhanced antibacterial effect on increasing its concentration without cellular toxicity and increasing the mechanical properties of composite resin. In addition, it does not affect the composite color, thus causing no threat to aesthetics. The antibacterial effect observed in the present study is also in accordance with a previous study that concluded that adding AgNPs into composite resin in small amounts provides a better antibacterial effect than unmodified resin [38]. Lower concentration showed lower toxicity and good mechanical properties. Composite resins with 1% AgNPs provided an almost 50% reduction in growth against *S. mutans* and *E. faecalis*. They inhibited *S. mutans* and *E. faecalis* biofilm growth on composite resin surface, likely due to consistency and a small amount of NPs incorporation into composite resin (*p* < 0.05).

The current study shows that introducing DEAE-Dextran AgNPs significantly improved the compressive strength. Mechanical properties were enhanced by increasing the concentration in DEAE-Dextran AgNPs. The enhanced mechanical properties can be due to the enhanced blending capacity of the AgNPs upon coating with DEAE-Dextran [21]. This analysis indicates that adding DEAE-Dextran AgNPs to resin-based composite restorative materials improved mechanical qualities such as compressive strength.

### Limitations

In this research, only lower concentrations, i.e., 1, 2.5, and 5%, of DEAE-AgNPs in dental composites were tested to conserve mechanical properties. The antibacterial, mechanical, and biocompatibility testing of resin composites with DEAE-Dextran at higher concentrations need to be explored in the future.

## 5. Conclusions

DEAE-Dextran AgNPs were successfully prepared with enhanced mechanical properties that effectively inhibited *S. mutans* and *E. faecalis* strains and polymicrobial biofilms when incorporated in dental composites. DEAE-Dextran coating on AgNPs limited aggregation and enhanced the blending capacity of AgNPs in dental resin composites. Adding these NPs will prevent polymerization shrinkage and address micro-crack formation that leads to secondary caries while making no compromise on esthetics. DEAE-Dextran AgNPs can be tested for their effectiveness as an antibacterial agent in dental adhesives in future research.

## Data Availability

Not applicable.
